# Targeting Oncogenic BRAF: Past, Present, and Future

**DOI:** 10.3390/cancers11081197

**Published:** 2019-08-16

**Authors:** Aubhishek Zaman, Wei Wu, Trever G. Bivona

**Affiliations:** 1 Department of Medicine, University of California, San Francisco, CA 94143, USA; 2 Helen Diller Family Comprehensive Cancer Center, University of California, San Francisco, CA 94158, USA

**Keywords:** BRAF, oncogene, precision medicine, targeted therapy, drug resistance

## Abstract

Identifying recurrent somatic genetic alterations of, and dependency on, the kinase BRAF has enabled a “precision medicine” paradigm to diagnose and treat BRAF-driven tumors. Although targeted kinase inhibitors against BRAF are effective in a subset of mutant BRAF tumors, resistance to the therapy inevitably emerges. In this review, we discuss BRAF biology, both in wild-type and mutant settings. We discuss the predominant BRAF mutations and we outline therapeutic strategies to block mutant BRAF and cancer growth. We highlight common mechanistic themes that underpin different classes of resistance mechanisms against BRAF-targeted therapies and discuss tumor heterogeneity and co-occurring molecular alterations as a potential source of therapy resistance. We outline promising therapy approaches to overcome these barriers to the long-term control of BRAF-driven tumors and emphasize how an extensive understanding of these themes can offer more pre-emptive, improved therapeutic strategies.

## 1. Introduction

During transformation, cancer cells accumulate many genetic and epigenetic alterations [1]. In the 1970s and 1980s, building on the discovery of the proto-oncogene Src and tumor suppressor Rb, many of such core molecular aberrations of cancers were identified [2,3]. Using this knowledge-driven approach, the targeting of oncogenic alterations, such as the gene rearrangement BCR-ABL in chronic myelogenous leukemia (CML), ushered in the current era of “precision medicine” and “targeted therapy” [4]. Vast improvements in technologies such as massively parallel DNA and RNA sequencing and computational analysis of large datasets have led to molecular profiling of thousands of tumors. Many multi-omics efforts have identified alterations in genomic and epigenomic features in tumors, such as DNA mutations, chromosomal copy number, coding and non-coding gene expression, promoter methylation, protein expression, and metabolic activity. A systematic analysis of cancer -omics analysis is now the standard for modern cancer diagnosis, and -omics-informed targeted therapy has become a key component of precision oncology, moving the field beyond general cytotoxic chemotherapy in many tumor types [5].

The Cancer Genome Atlas (TCGA) is a large-scale multi-omics effort that has led to comprehensive analysis and identification of novel molecular alterations, which not only classify tumors into distinct subclasses for therapy but also predict therapy response [6,7]. So far, a range of different oncogenes and tumor suppressors have been identified in different types of cancer [8]. For example, in breast cancer, *HER2, PIK3CA*, and *GATA3* were shown to have driver somatic mutations with incidence rates over 10%. Pancreatic cancer, on the other hand, showed a remarkable prevalence of *KRAS* mutations (>90%) [9]. *PIK3CA* was also found frequently mutated in small-cell lung cancer (SCLC) [10]. On the other hand, in non-small cell lung cancer (NSCLC), a staggering ≈60% of the tumors had alterations in chief “driver oncogenes” including *EGFR, BRAF*, and *KRAS* combined [11,12,13]. TCGA analysis also indicated a majority of thyroid cancers to be RAS or BRAF mutant [14]. Additionally, the analyses from “Pan Cancer Atlas” projects also reinforced the presence of frequent molecular alterations in previously identified major tumor suppressor genes (TSGs) such as *TP53*, *BRCA1/2*, *CDKN2A/B*, *PTEN*, and *NF1*. However, the crosstalk amongst these cataloged oncogenes and tumor suppressor genes, especially in the context of tumor heterogeneity and evolution, remains to be completely understood [8,12].

Cancer cells harbor thousands of non-synonymous mutations. Yet, only a subset of these alterations are considered as driver mutations due to their active role(s) in cancer development and progression; the rest are considered as coincidental passenger mutations that are dispensable for cancer cell viability and are accrued during the course of tumor evolution [15,16,17]. Mutant *BRAF* is a bona fide “driver oncogene”, since mutant BRAF inactivation often induces cancer cell toxicity, which indicates the establishment of an acquired dependency of tumor cells on oncogenic, mutant forms of BRAF [18]. Targeted inactivation of BRAF and similar driver oncogenes, which are often protein kinases, by pharmacologic inhibitors is an archetypal example of targeted therapeutic intervention in cancers [18,19].

Recently, a number of multi-omics studies have revealed not only the heterogeneity and complexities of the cancer genomic landscape, but also the dynamic nature of the therapy-driven tumor evolution. It is now appreciated that tumor cells are heterogeneous in terms of many features, including genetic mutation, transcriptional regulation, and signal transduction events and outputs. Furthermore, it has become increasingly clear that therapy-induced tumor evolution follows both Darwinian and Lamarckian evolutionary routes to give rise to resistance programs that bypass the therapy [20,21]. Hence, a comprehensive understanding of tumor biology has become a crucial prerequisite for predicting the therapy-driven evolutionary trajectory of treatment-naïve tumors and the successful implementation of novel cancer therapeutics. In this review, we discuss BRAF biology and the signaling consequences of mutant BRAF in tumors. We also summarize recent developments of, and prospects for, genomics-guided personalized therapy approaches in BRAF-mutant tumors in the light of the complexities of tumor biology and evolution.

## 2. BRAF Biology

In response to external stimuli (e.g., growth factors) that activate receptors such as receptor tyrosine kinases (RTK), RAS GTPases relay signal transduction to the mitogen-activated protein kinase (MAPK)/extracellular signal-regulated kinase (ERK) cascade [22]. The three-tier kinase cascade that constitutes MAPK/ERK signaling is one of the most well studied oncogenic signaling pathways, and the hierarchical composition of the signaling cascade includes MAPKKK (e.g., BRAF), MAPKK (e.g., MEK), and MAPK (e.g., ERK1/2). Activation of this signaling pathway results in a wide range of effector events leading to transcriptional rewiring and cellular growth signaling [23]. Importantly, in normal conditions, a range of negative regulators of ERK signaling are direct downstream targets of ERK output; such negative feedback loops ensure that the duration and magnitude of pathway signaling are appropriate for normal physiological states [23]. For example, one class of ERK target genes is the dual-specificity phosphatase family proteins (DUSPs) that bind directly to ERK and negatively regulate ERK activity [24]. Likewise, another ERK target class, Sprouty proteins (SPRYs), bind to upstream activating adapter protein complexes of MAPK signaling, thereby negatively regulating MAPK signaling output [25]. Fine tuning of this signaling regulation is crucial for maintaining normal homeostasis and growth signaling. The physiological regulation of the MAPK signaling becomes dysregulated in many pathogenic disease contexts, such as cancer [26].

In mammalian cells, there are three RAF proteins, namely, ARAF, BRAF, and CRAF (also known as RAF1). RAF family members are composed of three conserved domains—conserved regions 1, 2, 3 (CR1, CR2, CR3), respectively. CR1 is a RAS GTP-binding self-regulatory domain, CR2 is a serine-rich hinge region, and CR3 is a catalytic serine/threonine protein kinase domain that phosphorylates a consensus sequence on protein substrates [27,28,29]. Although all three RAF kinases play important roles in normal physiology, BRAF is the predominant RAF kinase that is altered in many different cancer types; for example, almost 60% of melanomas, 60% of thyroid cancers, 15% of colorectal cancers, and 5–8% of non-small cell lung cancers (NSCLCs) show *BRAF* mutations [30]. The biological specificity of *BRAF* (and not other RAFs) as an oncogene is not completely understood. Possible explanations are BRAF’s tissue- and context-specific nature of regulation and/or expression levels and/or its ability to activate MEK, compared to CRAF and ARAF that may function more broadly as “housekeeping” genes [31]. For these reasons, we focus on the role of *BRAF* as an oncogene.

In its active form, BRAF forms a dimer and functions as a serine/threonine-specific protein kinase. The BRAF N-terminal regulatory domain, when inactive, autoinhibits the C-terminal kinase domain. These dimers are further stabilized by 14-3-3 protein heterodimers [29]. When activated by RAS GTPases, RAF is recruited to the plasma membrane. This is regulated by RAS–GTP interaction and a suite of differential phosphorylation events on RAF proteins [32]. The BRAF protein contains 766 amino acids; its CR1 region spans the range of 120–280 amino acids. CR1 autoinhibits the CR3 kinase domain of BRAF to protect cells from misfiring inappropriate kinase substrate reaction kinetics. Amino acids 155–227 constitute the RAS-binding domain (RBD). Upon RAS-GTP binding, this region releases the autoinhibitory CR1–CR3 interaction to free CR1 and cease kinase inhibition. Amino acids 234–280 constitute a phorbol ester (diacylglycerol)-binding zinc finger motif that participates in RAF membrane docking after RAS-GTP binding. CR2, on the other hand, functions as a hinge for CR1 and CR3. CR3, a dual lobe structure, contains the kinase domain and spans the range of 457–717 amino acids [33,34].

The smaller N-terminal lobe binds ATP, whereas the C-terminal lobe binds substrate proteins. The two lobes flank the kinase active cleft with the active residue D576 facing the inside of the cleft. The N-terminal lobe also contains the P-loop that stabilizes ATP-binding through electrostatic interaction with the negatively charged phosphate group. Hydrophobic interactions stabilize interaction with the ATP nucleoside [33].

## 3. Mutant BRAF: A Prototype Driver Oncogene

**Figure 1:**
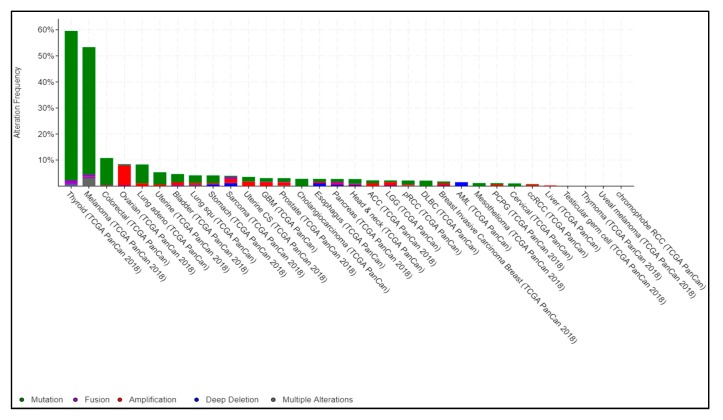
Pan-cancer BRAF alterations from The Cancer Genome Atlas (TCGA). TCGA-generated pan-cancer alteration frequency of BRAF was extracted from cBioportal (https://www.cbioportal.org). Data are represented as a stacked histogram plot. Colors represent different types of alterations as indicated in the legend.

Mutated *BRAF* is a major driver gene alteration in cancers of multiple tissue origins. Almost 60% of melanomas are reported to be BRAF mutant, and mutations in this gene are also present in non-Hodgkin’s lymphoma, colorectal cancer, papillary thyroid carcinoma, NSCLC, glioblastoma as well as inflammatory diseases [35,36]. In papillary thyroid carcinoma, 60% of cases show activating somatic alterations of genes encoding effectors in the MAPK signaling pathway, including BRAF and RAS. In human colorectal cancers, 46% of cases are BRAF mutant. In NSCLC, *BRAF* mutation has also been shown to be a recurrent alteration. Importantly, targeted perturbation of mutant BRAF is effective in many patients with BRAF-mutant (V600) alleles (Figure 1). Taken together, these observations establish *BRAF* as a key driver gene that is often altered in many different cancers [9].

**Figure 2:**
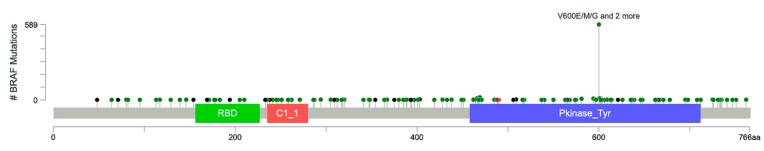
BRAF mutation spectrum in cancer. TCGA pan-cancer studies from cBioportal (https://www.cbioportal.org) were used for BRAF mutation lollipop plot. This plot summarizes 32 studies from TCGA that constitute 10,953 patients/10,967 samples. Somatic BRAF mutation frequency is 7.0% in these samples. The diagram shows the backbone of BRAF protein containing 766 amino acids (aa) with three main domains: (1) Raf-like RAS-binding domain (RBD, that spans 156–227 amino acids green), (2) phorbol esters/diacylglcerol-binding domain (C1 domain, 235–280aa, red), and (3) protein tyrosine kinase domain (Pkinase_Tyr, 458–712 aa, blue). The circles with different colors represent types of mutations: dark green, missense mutations; black, truncating mutations (including nonsense, nonstop, frameshift deletion, frameshift insertion, splice site mutations); dark red, in-frame deletion, in-frame insertion; pink, other mutations. *Y*-axis shows the frequency of particular BRAF mutations.

**Figure 3:**
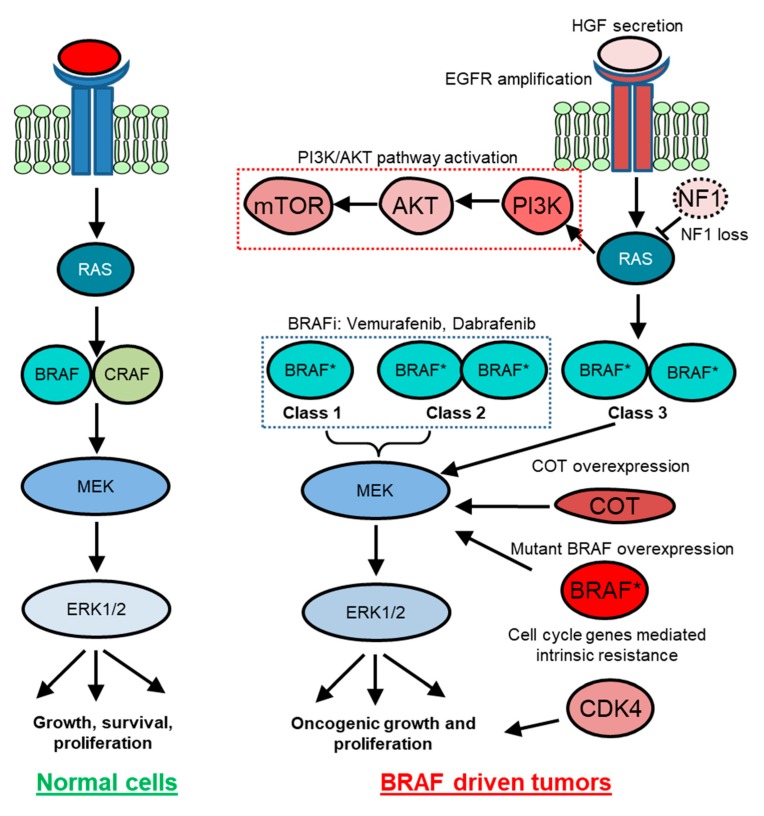
BRAF-mediated signaling in normal and cancer cells. In normal cells, external growth stimuli activate receptor tyrosine kinase (RTK) and RAS, which relays growth signals to the mitogen-activated protein kinase (MAPK) pathway kinase cascade. In BRAF-driven cancers, mutant BRAF (BRAF *) can either act RAS independently as a monomer (Class 1) and as a dimer (Class 2) or act RAS dependently (Class 3) to hyperactivate cellular growth. Class 1 and Class 2 tumors respond to BRAF inhibitor-targeted therapy. However, various intrinsic or adaptive resistance mechanisms attenuate response to targeted BRAF inactivation. For example, preexisting NF1 loss, CDK4 mutations, and increased COT and YAP expression may specify intrinsic resistance. Therapy-induced HGF secretion and PI3K-AKT pathway activation are examples of some of the adaptive resistance mechanisms. On the other hand, EGFR-amplified population gives rise to acquired resistance to BRAF inhibitors.

Approximately 200 BRAF-mutant alleles have been identified in human tumors (Figure 2). So far, almost 30 distinct mutations of BRAF have been functionally characterized [37]. Interestingly, BRAF mutations can be categorized into three classes based on their effect on the activity of BRAF (Table 1). Class 1 BRAF mutations are RAS-independent and function as an active monomer. On the contrary, Class 2 mutant BRAF functions as an active dimer. Both Class 1 and Class 2 mutant BRAF proteins become largely independent from their upstream regulator RAS GTPases for activity. This uncoupling results in constitutive activation of BRAF that is independent of upstream stimuli for growth and proliferation in cancer. In contrast, Class 3 mutant BRAF proteins depend on RAS signaling for optimum activation (Figure 2 and Figure 3) [38]. These mutants are kinase impaired and show higher MAPK signaling output due to RAS activation, for mechanistic reasons that are poorly defined. Hence, Class 3 mutant BRAF activity is dependent on RAS-GTP levels. Thus, the blockade of upstream RAS signaling is a potential therapeutic strategy for Class 3 BRAF-mutant tumors, in contrast to Class 1 or 2 BRAF mutants [37,38,39].

This concept is also relevant in the context of targeted therapy drug resistance phenotypes. Since MAPK signaling output requires RAS-induced RAF dimerization and is autoinhibited by feedback repression, activated BRAF mutants can bypass this repression broadly through acquiring Class 1 BRAF V600E mutations that are sufficient to function as hyperactive monomers in a RAS dispensable manner [40,41].

Recent publications have also shown that oncogenic BRAF gene fusions can also activate BRAF in melanomas and other cancers [42,43,44]. In these cases, the BRAF kinase domain is fused with N-terminal partner genes such as SOX10, AGK, SEPT3. The fusions result in an alteration of the BRAF copy number and activity independent of common missense BRAF mutations. However, there were significant differences in phenotypes, clinical responses, and mechanisms by which these oncogenic BRAF forms operate, and this is an area of current investigation [43,44].

## 4. Targeting Oncogenic BRAF

A better understanding of BRAF biology and an improved classification of the BRAF gene alteration spectrum in cancer enabled the identification and design of small molecule inhibitors that specifically inactivate the catalytic activity of BRAF. These pharmacological developments and their iterative improvements have not only been clinically relevant, but have also informed the understanding of physiologic RAF regulation. Several ATP competitive and allosteric inhibitors that bind and perturb catalytic kinase activity of BRAF have been clinically investigated. Sorafenib was the first BRAF inhibitor developed and was clinically evaluated in melanomas. It is a broad-spectrum kinase inhibitor and targeted wild-type BRAF, mutant BRAF^V600E^, and CRAF, as well as VEGFR, PDGFR, CKIT, and FLT3. Determining optimal dosing for this drug proved difficult due to the lack of mutant specificity and the preponderance of targets. Sorafenib proved to be ineffectual for the treatment of melanoma as both a single agent and in combination with chemotherapy [45].

Vemurafenib (PLX4032), one of the first mutant-specific BRAF inhibitors, is specific for BRAF^V600E^ [46,47]. It was initially approved by the U.S. Food and Drug Administration (FDA) for advanced-stage melanoma treatment, and in Phase I clinical trials treatment with vemurafenib induced disease stabilization, as well as some cases of marked regression of BRAF^V600E^ melanomas. In addition, the Phase II clinical trial demonstrated an increase in the objective response rate (ORR) and the average duration of response in the vemurafenib arm. The Phase III clinical trial reported 5.3-month median progression-free survival (PFS) in the vemurafenib treatment group, compared with a 1.6-month median PFS in the control treatment group. Dabrafenib, another mutant BRAF inhibitor, attained FDA approval in 2013. Phase III clinical trial results for this compound marked an increase in ORR and PFS. These observations were further recapitulated in classical hairy cell carcinoma that contains BRAF^V600E^ [48].

In BRAF-mutant colorectal and thyroid cancers, RAF inhibitors did not show much clinical effect as single agents [46,47]. Additionally, certain patients with mutant BRAF-driven tumors showed evidence of paradoxical activation of MAPK pathway upon BRAF inhibition [49]. These patients manifested signs of cutaneous lesions with squamous cell carcinoma histology. In these patients, secondary mutations in HRAS were observed and attributed as a mechanism that potentiated BRAF inhibitor-induced paradoxical activation of the MAPK pathway to induce these secondary skin cancers. In addition to this liability, the durability of BRAF inhibitor monotherapy treatment is limited by drug resistance [49].

MEK inhibitors have also been extensively investigated in BRAF-mutant and other MAPK pathway-driven tumors [50]. In melanoma, Phase I and II trials of the MEK inhibitor trametinib showed improvement in both PFS and overall survival (OS) [51]. In BRAF-mutant melanoma, a second MEK inhibitor MEK162 showed similar responses [52]. MEK inhibitors did not show the paradoxical MAPK pathway activation response observed with vemurafenib and dabrafenib. However, the efficacy of MEK inhibitors as single agents is relatively modest, whereas the combination of BRAF and MEK inhibitors in BRAF-mutant cancers has shown great success [53,54]. Therefore, BRAF and MEK inhibitor combinatorial treatments, in the form of trametinib + dabrafenib and cobimetinib + vemurafenib, were approved by the FDA for patients with advanced BRAF^V600E^ melanoma [55,56]. BRAF and MEK inhibitor combination therapy in treatment-naïve patients showed better ORR than BRAF inhibitor monotherapy. In metastatic melanoma, the dabrafenib and trametinib combination treatment showed a median OS of 27.4 months compared to 17.4 months for dabrafenib alone [55,56]. These observations led to the adoption of RAF and MEK dual inhibition as a standard of care for these patients.

These observations have also given rise to similar treatment strategies for BRAF-mutant NSCLC. In 2017, Planchard et al. reported that the Phase II clinical trial of dabrafenib and trametinib combination treatment achieved an impressive 64% ORR [57]. The most common BRAF mutation in lung cancer is the BRAF V600E mutation, accounting for roughly 50% of BRAF-mutant NSCLC [58]. These results led to the 2017 FDA approval of the combination of dabrafenib and trametinib for advanced NSCLC harboring the BRAF V600E mutation.

In metastatic colorectal cancer, Corcoran et al. showed that dabrafenib and trametinib combination achieved partial or complete response in a subset of 12% (5 out of 43) patients [59]. In a follow-up study, they also showed that combinatorial inactivation of BRAF, MEK, and EGFR (using anti-EGFR antibody panitumumab) achieves a higher response rate of 21% (19 out of 91) in BRAF^V600E^-positive patients [60]. These clinical successes were facilitated by the mechanistic work that suggested the role of EGFR-mediated MAPK reactivation in certain BRAF inhibitor-treated BRAF^V600E^-positive tumors [41,61,62].

Because BRAF is the immediate proximal regulatory component of the RAS signaling network and because MAPK pathway activation is a bona fide oncogenic feature in neoplastic cells, BRAF inactivation could address a wide variety of MAPK pathway-driven cancers [38,63]. However, the mechanisms of BRAF activity regulation are now recognized to be more complex than anticipated. For example, accumulating evidence indicates a requirement of RAF dimerization for RAS-dependent RAF kinase activation in normal cells. In this context, although BRAF can dimerize with any of the RAF family members, BRAF-CRAF heterodimers are thought to predominate (Figure 3) [64,65]. In cancer, dysfunctional regulation is observed: (1) mutant BRAF can often execute a signaling function independent of RAS; (2) V600-mutant BRAF proteins can carry out downstream signaling as monomers; and (3) RAF proteins can homodimerize. Based on these principles, paradoxical activation of MAPK signaling can occur upon BRAF inactivation in RAS mutant tumors. This often results from an unexpected allosteric stabilization of BRAF-CRAF heterodimers [66]. Consistent with this, BRAF dimerization has been reported to modulate targeted therapy response. The Rosen group further demonstrated that ATP competitive inhibitors that target the BRAF kinase activity seem to work on monomeric mutant BRAF (e.g., V600E) but had little or no effect on mutant BRAF proteins that form dimers. Their mechanistic work indicated that the binding of the inhibitors at the kinase domain of the dimerization proficient BRAF mutants results in conformational changes leading to lower affinity of the compound on the second site binding. In 2015, Zhang et al. demonstrated that pharmacologic inhibitors (so called “paradox breakers”), which bind to RAF protomers and selectively disrupt dimer formation, can block the paradoxical MAPK activation [67]. These observations resulted in a rejuvenated interest in clinically potent dimerization domain inhibitors as a therapeutic strategy [68]. Moreover, for Class 3 BRAF-mutant tumors, which are RAS dependent, recent studies have indicated that the use of SHP2 inhibitors holds an exciting therapeutic promise in part because SHP2 inhibition reduces upstream signaling that promotes RAS-GTP and RAF dimerization [38,69]

## 5. Resistance to BRAF Inhibitor Therapy and Strategies to Confront Resistance

Despite remarkable early remission and overall improvement of patient outcome, resistance to RAF/MEK-targeted therapies invariably occurs [5,70,71]. Resistance to therapy can be classified into three categories as intrinsic resistance, adaptive resistance, and acquired resistance [49]. Intrinsic resistance indicates the lack of response to initial treatment. For example, tumors harboring BRAF^V600E^ failed to respond to vemurafenib and trametinib due to additional preexisting copy number amplification of cyclin D1 and elevated YAP1 expression [72,73,74]. Conversely, resistance against targeted RAF/MEK inactivation can also occur due to a de novo adaptation of cellular epigenetic and transcription programs leading to adaptive resistance and a partial response to the therapy [75]. For example, recent publications indicate that adaptive upregulation of NFκB program mediates MAPK pathway inhibitor resistance [76]. Acquired resistance arises due to the selective pressure imposed by therapy onto tumor cells consisting of heterogeneous genetic alterations that are selected out and/or due to the acquisition of therapy-induced de novo alterations [77]. Acquired resistance via the activation of parallel signaling pathways (such as RTKs) and “on-target” accumulation of mutations in the BRAF pathway itself are some examples of acquired resistance [40,78]. Although the existence of biological overlap amongst these classes is apparent, detailed mechanistic insights underlying therapy-induced state transitions in cancer are lacking.

The incomplete and short-lived nature of targeted therapy response highlighted the concept of a residual disease state in tumors [79]. The residual disease state is not eliminated by the targeted therapy and serves as a prelude for subsequent tumor progression and acquired resistance. A growing body of literature indicates that this evolution of tumors through these different stages is dictated by a heterogeneous combination of multiple molecular drivers and cell states that reflect profound plasticity [80,81]. There remains an open question as to how to best identify intra- and inter-tumor heterogeneity of BRAF-mutant cancers and how to best account for it in order to strategize improved treatment options. So-called “liquid biopsies” are one approach that is under investigation to address this gap [82,83,84].

### 5.1. Intrinsic Resistance

In this section, we discuss examples of intrinsic resistance mechanisms in BRAF-mutant tumors. Cell-cycle gene alterations are one such example. Using patient-derived cell lines, Smalley et al. demonstrated that co-occurring mutations in cyclin dependent kinase 4 (CDK4) resists response of BRAF inactivation in BRAF-mutant cells [85]. Consistent with these findings, aberrations in cell cycle-related genes are found in patient cohorts who are non-responders to BRAF inhibitor treatment. CCND1 amplification is noted in 15–20% of BRAF-mutant melanomas and can contribute to therapy resistance (Figure 3) [85].

Loss of function of the tumor suppressor PTEN also predicts poor prognosis upon BRAF inhibitor treatment [86]. Nathanson et al. reported that in treatment-naïve melanoma, baseline PTEN loss/mutation showed a trend for shorter median PFS—18.3 weeks (PTEN mutant) versus 32.1 weeks (PTEN wild type) [86]. PTEN regulates the phosphotidylinositol-3-kinase (PI3K) pathway, which is a well-studied oncogenic pathway in multiple cancer types These observations have invigorated PI3K and BRAF dual inhibition strategies in patients with PTEN null melanoma [86]. Accumulating evidence also points towards high growth factor signaling as a mechanism of intrinsic resistance against BRAF inactivation. For example, using a co-culture model for stromal cells and tumor cells, Wilson et al. showed that hepatocyte growth factor (HGF) and its interaction with its receptor, CMET, mediate intrinsic resistance to BRAF inhibition [87].

MAPK pathway alterations are also implicated in resistance in BRAF-driven tumors. In a malignant melanoma preclinical model, Johannessen et al. [88] showed that an elevated copy number of MAP3K8 (also known as COT) specifies BRAF inhibitor resistance through MAPK pathway reactivation. In a separate study, using a pooled shRNA screen on patient-derived cell lines, Whittaker et al. showed that loss of the negative regulators of RAS/MAPK signaling, NF1 mediates resistance to RAF and MEK inhibitors through sustained MAPK pathway activation (Figure 3) [89]. Moreover, Hodis et al. and Krauthammer et al. also indicated RAC1 gain of function alterations as an intrinsic resistance mechanism [90,91]. Their work put forth a model where preexisting pools of high RAC1 activity cells were selected for drug resistance [92].

Intrinsic resistance against MAPK inhibitors is also mediated by the Hippo pathway effector, YAP [74]. YAP is a transcriptional co-activator. Hippo pathway activation causes cytoplasmic sequestration and/or proteolytic degradation of YAP leading to the attenuation of YAP-mediated transcriptional program. Genetic screening approaches found YAP as a mediator for BRAF inhibitor resistance. Lin et al. demonstrated that high YAP1 expression was associated with resistance to BRAF inhibitor treatment in preclinical models and with poor survival in a cohort of patients with melanoma and NSCLC treated with BRAF inhibitors. This resistance to BRAF inhibition could be reversed by genetic inhibition of YAP in preclinical models (Figure 3) [74]. YAP has also been reported as an intrinsic resistance mechanism in a cohort of KRAS-mutant NSCLC and melanoma [93,94]. Whether YAP-mediated resistance against BRAF-targeted therapy may function as an adaptive response, in addition to intrinsic resistance, is unclear [73].

### 5.2. Adaptive Resistance

Transcriptional and epigenetic rewiring is another common resistance mechanism that operates as an adaptive mode of drug resistance [95]. For example, adaptive overexpression of stromal HGF secretion and underexpression of CTLA4, BIM, and antigen presentation genes (B2M, HLA-A, HLA-B, and TAP1) have been cited as mechanisms of tumor resistance upon BRAF inactivation [75,96,97,98,99,100,101,102,103]. Furthermore, under physiologic conditions, MAPK signaling activation activates a negative feedback loop that attenuates standard RTK signaling. Thus, BRAF-mutant tumors have a baseline low level of these RTK activities and BRAF inhibition in these tumors has been reported to cause de-repression of RTK signaling. This can occur either through the relief of inhibitory protein–protein interactions or the secretion of RTK ligands [104]. Further study into this category of resistance is warranted.

### 5.3. Acquired Resistance

Most acquired resistance mechanisms in BRAF-mutant tumors are through reactivation of the MAPK pathway [75] (Table 2). For example, mutational activation of NRAS and MEK1/2 and increased CRAF protein level have been reported in the presence of BRAF inhibition in BRAF-mutant melanoma. Moreover, overexpression of BRAF^V600E^ and splicing variants of BRAF have been observed in melanoma or lung cancer upon acquired resistance (Figure 3) [49,75,105]. BRAF^V600E^ amplification has also been observed in this context [49,75].

Almost one third of the acquired resistance cases occur in a MAPK pathway-independent manner [114]. Examples of acquired resistance mechanisms to MAPK pathway inactivation that are not solely operative via MAPK pathway reactivation are MITF, WNT-signaling (LEF1, FZD6, WNT11, and WNT10A), and RTK amplifications and/or hyperactivation (e.g., MET, AXL, EGFR, HER2, HER3, FGFR) [40,88,96,102,115,116,117]. When comparing treatment-naïve and dabrafenib- or vemurafenib-treated melanoma patients, Johnson et al. identified PI3K-AKT signaling and increased expression of PDGFRβ or insulin-like growth factor 1 receptor (IGF1R) as mechanisms of therapy resistance [78,99,106]. Amplification of MITF, a melanoma lineage-specific transcription factor, was shown to promote acquired resistance to BRAF inhibition. MITF amplification mediated overexpression of the anti-apoptotic protein BCL2-related protein A1 (BCL2A1), which in turn caused acquired resistance [115].

### 5.4. Therapeutic Strategies Against Resistance

Due to resistance mechanisms, BRAF-targeted therapy has not achieved durable survival benefits as a single agent [118,119]. Because resistance frequently occurs via MAPK pathway re-activation, MEK inhibitor plus BRAF inhibitor combinations gained interest, as discussed above [120]. This approach has improved outcomes for patients, although resistance to the combination therapy remains a problem and the mechanisms of resistance are under investigation [121,122].

Different treatment paradigms varying drug dose and schedules are under investigation to prolong therapeutic response [49,123]. Prolonged continuous targeting may intensify the emergence of resistance mechanisms and carries the risk of continuous drug toxicity; intermittent targeting may allow for the mutant BRAF subpopulation of cells to regrow to induce drug-resistant tumor progression. In this regard, mechanistic studies have shown that normal cells require intermediate levels of RAF–MEK–ERK pathway activation for optimum proliferative effect [124,125,126]. Intriguingly, it was demonstrated that vemurafenib-resistant cells become dependent on the presence of the drug itself and hence drug withdrawal itself was sufficient for the regression of the tumor [124,125,127]. This observation has led to clinical trial design to test the efficacy of intermittent dosing to forestall the onset of drug-resistant disease.

Furthermore, mechanistic studies indicate that vertical blockade of MAPK signaling by BRAF inhibitors in combination with MEK inhibitors holds untapped therapeutic opportunities for class-specific polytherapy [127,128]. A recent publication from Xue et al. [122] indicated that sequential monotherapy results in the selection of BRAF-amplified cells that eventually cause acquired resistance to emerge. In contrast, vertical blockade of ERK through polytherapy suppresses this evolutionary trajectory by mounting a sufficient fitness barrier for the BRAF-amplified clonal population. Interestingly, the findings indicated that an intermittent scheduling of polytherapy was also able to block treatment-induced parallel evolution of the BRAF amplification. These observations indicate an intermittent dosing of polytherapy may be required to perturb tumor evolution on all fronts [122]. Consistent with this, similar vertical blockade strategy was adopted in a Phase II clinical trial of metastatic colon cancer where EGFR, BRAF, and MEK were co-inhibited. The results indicated not only a superior ORR but also PFS and OS, as described above [60,61]. Moreover, recent studies have shown that SHP2 inhibitors were effective in bypassing paradoxical activation of MAPK signaling upon BRAF inhibition in RAS-mutant cancers [69]. These studies highlight the importance of molecular classification and profiling to better predict therapy regimen.

## 6. Summary, Challenges, and Prospects

**Figure 4:**
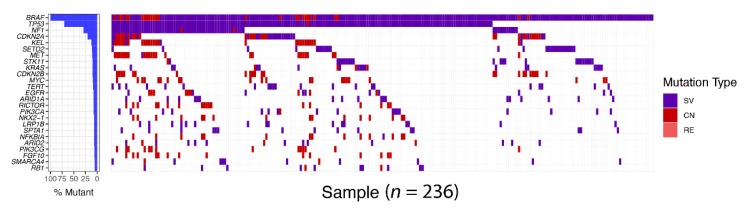
BRAF alterations concurrent with other genetic aberrations in non-small cell lung cancer (NSCLC). Targeted tumor exome sequencing using 403 cancer gene panel was performed by Foundation medicine. In all, 236 samples with BRAF alterations, such as structural variation (SV), copy number variation (CV), and rearrangement (RE), were stratified from 30,000 non-small cell lung cancer samples. Oncoprint of BRAF-mutant tumor shows coexistence of other genetic alterations with BRAF gene alteration. The different frequency of gene alterations is indicated on the left bar plot.

Complete and durable responses to precision medicine or targeted therapy are rare in individuals with advanced-stage solid cancers such as melanoma and lung cancer. A profound initial response is inevitably followed by resistance to therapeutic agents. Accumulating evidence suggests that tumor heterogeneity at multiple levels plays an important role in the incomplete and short-lived nature of targeted therapy efficacy. Targeted therapy may show an antiproliferative effect on a pool of cells. Cells unaffected by the therapy flourish and ultimately form the bulk of the tumor. These observations indicate that understanding tumor heterogeneity is crucial for defining treatment-mediated tumor evolution that ultimately leads to resistance. The presence of co-occurring modifiers, which during driver oncoprotein inhibition give rise to resistance programs, reveal the multi-faceted and heterogeneous progression that characterizes tumor evolution (Figure 4). Hence, much effort is being expended to better understand tumor heterogeneity and account for it during initial treatment. Indeed, increased baseline genetic heterogeneity correlated with a shorter duration of response to EGFR inhibitor therapy [81,129]. Through genomic analysis of 1122 EGFR-mutant lung cancer cell free DNA samples, Blakely et al. demonstrated that tumor genomic complexity increased over time [80]. Hence, upfront polytherapy with a cocktail of drugs to perturb both oncogenic programs as well as the resistance programs is a promising approach (as described above), and similar studies in BRAF-mutant tumors are lacking.

Moreover, adaptive resistance to RAF inhibition has also been associated with the presence of a subpopulation of slowly dividing “stem-like” cells [130,131]. In BRAF-mutant cancers, these “stem-like” cells can tolerate otherwise toxic levels of MAPK perturbation by activating a cellular de-differentiation program through epigenetic modifications [130,132]. These observations have resulted in an ongoing research focus that aims to co-target epigenetic remodeling and MAPK pathways in BRAF-mutant tumors [133].

Recently, immunotherapeutic approaches have also been considered as a component of the treatment of BRAF-mutant cancers [134]. Although targeted BRAF inactivation results in a more robust initial response than immunotherapies in most patients, immunotherapy with antibodies against programmed death 1 (PD-1) produces more durable responses [135,136]. PD1 and PD-L1 are surface receptors that attenuate the T cell-mediated killing of tumor cells and thereby act as immune checkpoint molecules. For patients with BRAF-mutant metastatic melanoma, it is unclear whether immunotherapy or kinase inhibitors against BRAF and MEK should be applied in the first-line setting. In this regard, combining immunotherapy with kinase inhibitor therapy has opened up previously untapped opportunities. Ribas et al. showed that the combined application of MEK inhibitor and PD1 blockade resulted in encouraging responses in a subset of melanoma patients [135]. Additionally, the sequential application of these therapeutic options has also been proposed. For example, the prospect of immunotherapy as a consolidation therapy following chemotherapy or targeted kinase-inhibitor therapy is promising in advanced-stage NSCLC [136].

Mutations in the IFN-γ pathway, activation of bypass signaling via the TGF-β and PI3K/mTOR pathways, and loss of STK11 have been reported in association with immunotherapy resistance [137,138,139,140]. Hence, further research is required to systematically map tumor evolution upon immunotherapeutic interventions and synergies between MAPK pathway inhibition and checkpoint blockade.

A promise of precision oncology is that the genomic-scale understanding of BRAF-mutant tumors, both at baseline and longitudinally during treatment, will allow for predicting tumor evolutionary trajectories at a higher resolution. Better characterization, classification and profiling of therapy-induced tumor evolution should yield therapeutic options that incorporate knowledge of cancer cell and tumor microenvironment cell factors to preclude or constrain tumor evolution during initial treatment and transform BRAF-mutant cancers into chronic or curable conditions.

## Figures and Tables

**Table 1:** Catalog of prominent BRAF mutations in cancer.

Class	RAS Dependent	Missense Mutations
**Class 1**	No	V600E, V600K, V600D, V600R, V600M, etc.
**Class 2**	No	K601E, K601N, K601T, L597Q, L597V, G469A, G469V, G469R, G464V, etc.
**Class 3**	Yes	D287H, V459L, G466V, G466E, G466A, S467L, G469E, N581S, N581I, D594N, D594G, D594A, D594H, F595L, G596D, etc.

**Table 2:** List of MAPK reactivation-mediated resistance mechanisms.

Resistance Mechanism	Molecular Details	Salient References
BRAF Amplification	BRAFV600E/K amplification	[40,78,97,98,106]
CRAF Overexpression	Transcriptional activation	[97,105,107]
BRAF Splice Variant and Truncation	Δexon 2–10, Δexon 2–8, Δexon 4–8, etc.	[40,78,106,107]
NRAS Mutation	G13R, Q61K mutations	[78,98,108,109]
MEK1 Mutation	K57E, I111S, P124S, G176S, E203K, etc., mutations	[78,99,110,111,112]
MEK2 Mutation	F57C, Q60P mutations	[78,99,111,112,113]
IGF1R Overexpression	Transcriptional activation	[78,98,110]
